# The role of detours in individual human navigation patterns of complex networks

**DOI:** 10.1038/s41598-020-57856-4

**Published:** 2020-01-24

**Authors:** András Gulyás, József Bíró, Gábor Rétvári, Márton Novák, Attila Kőrösi, Mariann Slíz, Zalán Heszberger

**Affiliations:** 1 MTA-BME Information Systems Research Group, Budapest University of Technology and Economics, Magyar tudósok krt. 2, H-1117 Budapest, Hungary; 2 Eöotvös Loránd University, Institute of Hungarian Linguistics and Finno-Ugric Studies, Múzeum krt. 4/A, H-1088 Budapest, Hungary

**Keywords:** Cognitive neuroscience, Scientific data

## Abstract

Despite its importance for public transportation, communication within organizations or the general understanding of organized knowledge, our understanding of how human individuals navigate complex networked systems is still limited owing to the lack of datasets recording a sufficient amount of navigation paths of individual humans. Here, we analyse 10587 paths recorded from 259 human subjects when navigating between nodes of a complex word-morph network. We find a clear presence of systematic detours organized around individual hierarchical scaffolds guiding navigation. Our dataset is the first enabling the visualization and analysis of scaffold hierarchies whose presence and role in supporting human navigation is assumed in existing navigational models. By using an information-theoretic argumentation, we argue that taking short detours following the hierarchical scaffolds is a clear sign of human subjects simplifying the interpretation of the complex networked system by an order of magnitude. We also discuss the role of these scaffolds in the phases of learning to navigate a network from scratch.

## Introduction

Everyday life is full of complex networked systems that humans recurringly navigate on a daily basis (e.g., travelling between locations in a city using public transportation). The available navigational datasets^1–4^ and models^3–10^ considering networked systems mostly target uncovering the average properties of a group of subjects and capture collective human behaviour. Moreover, in terms of human navigation, the existing experiments focus on the dynamic process of learning to navigate, i.e., how people incrementally learn an approximate map of the network. Thus, existing datasets do not have sufficient data or appropriate tracing methods permitting the analysis of long-term individual patterns. Here, we analyse the results of an experiment^11^ with human subjects solving navigational tasks in a complex word-morph network. The recorded average of 40.9 timely ordered paths from 259 subjects and more than 200 paths from 9 subjects makes the analysis of individual human navigation patterns possible. In contrast to existing studies, this amount of data enables the inference of characteristics about the steady-state way in which people choose a path between endpoints of a network *after* they have learned how to navigate the network in their individual way. We argue that this routine navigation process is more valuable to investigate since people use this approach on a daily basis. In the remainder of this study, we refer to this steady-state navigation simply as *human navigation*.

**Figure 1:**
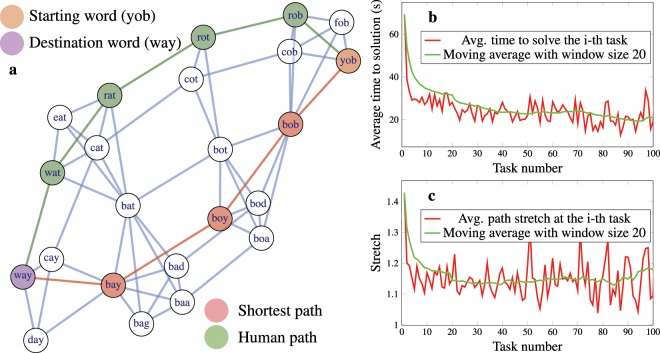
An example and high-level statistics of our navigation experiment. Panel (**a**) shows a sample section of the network of three-letter English words, in which two words are connected if they differ only in a single letter. When human subjects solve a navigation task, they come up with a path from a randomly given starting word to a destination word by changing only a single letter at each step such that they always obtain a valid intermediate English word. The red and green paths show a shortest and a slightly detoured human solution from “yob” to “way”. Panel (**b**) presents the average time it takes for human subjects to solve the n-th task in a row, while panel (**c**) shows the stretch of the human paths, i.e., the ratio of the length of the paths found by human subjects to the length of the shortest possible path in the word-morph network. While the average time to solve a task clearly decreases with the number of tasks solved, the stretch of the solutions stabilizes between 1.2 and 1.1. This suggests that human subjects develop a specific strategy in the first few rounds, but after a few tens of solved tasks, their strategy is not improved any further in terms of length. Therefore, they have a simplified interpretation of the network, and they find their paths through this, only slightly faster as time elapses.

The nodes of the word-morph network, from which we process the navigation paths, are English words that are connected if they differ in only a single letter. In this large and complex network, human subjects are given navigational tasks, i.e., to reach a destination word from a starting word by changing only one letter at a time, while still having meaningful words in intermediate states. Figure 1a shows a sample fragment of the word-morph network and two solutions (a shortest path and a human path) of the task with the starting word “yob” and destination word “way”.

Network theoreticians across many disciplines^12–16^ argue that the shortest path, i.e., the path containing the minimal number of intermediate steps in a network, between a source and a destination is a usable approximation of the real paths between them. In contrast with these results, our study shows that humans subjects frequently apply detours, even in the long run. Our main finding is that these detours are the consequences of how an individual interprets a complex networked system on its own level. We show that people tend to build up a significantly simpler representation of the word-morph network in the form of a hierarchy in their minds. A hierarchy is a way of interpreting an interconnection network by defining a central node (or a set of nodes) and referring to all other nodes with positions relative to, i.e., “above” or “below”, the central node. These hierarchies are then used as helper structures when forming the paths in the network. In this study, we will refer to these hierarchical helper structures simply as “scaffolds”.

As a result, real paths will be somewhat longer than the shortest alternatives, but the detours will be characteristic to the individual taking them, as no two individuals may abstract the same hierarchy of the network. Although there are existing models assuming latent hierarchical scaffolds aiding navigation^6,10,17–20^, this is the first study processing sufficient individual human navigation data to visualize and analyse these individually created hierarchies.

We discuss that navigational scaffold hierarchies may boost the learning process to navigate the word-morph network and reduce the memory requirement of navigation by an order of magnitude. Moreover, identifying the individual scaffold hierarchies as the enablers of memory-efficient navigation in the word-morph network is of particular importance since this may promote uncovering of navigational schemes in other complex networked systems considering not only humans. Similar detours have been identified in measurements capturing the collective behaviour in networks from diverse areas of life. Gao *et al*. showed that the paths of packets going through the internet are also detoured to a non-negligible extent^21^, and they showed that the hierarchical policies of internet packet routing may be responsible for a major proportion of the inflation. Detours have been identified in road networks by Zhu *et al*.^22^ and in cattle pen systems by Grandin^23^, while similar phenomena were also reported in airports^10,24^ and brain networks^10,25^.

## Results

For our study, we use data from an experiment with a word-morph game application for smartphones^26^ (see Methods for details). The application collected 19828 paths from 259 human subjects navigating the word-morph network, and the corresponding dataset was published in Scientific Data^11^. After cleaning the data from paths not referring to steady-state navigation, by removing tasks that were either unfinished, contained loops or took an extraordinarily long time (>300 seconds) to complete, our working dataset of paths was reduced to 10857 paths (for more details about data filtering, see Methods). The word-morph network is a complex network that is impossible for a human subject to keep fully in mind with its 1008 nodes and 8320 edges. The values of the average degree (i.e., the average number of edges emanating from the nodes), the diameter (the longest shortest path in the network) and the clustering coefficient^13^ of the network are 16.39, 9 and 0.44 respectively. To attain a high-level impression about the performance of human navigation, we have plotted the average time needed to solve the n-th task in a row in Fig. 1b. We can see that after a few initial rounds, human subjects find a solution in approximately 30 seconds on average, and from there on, they slowly improve to approximately 20 seconds after solving 100 tasks. Notably, it is an intrinsically astonishing finding that after a few rounds, people can find paths in this complex maze very efficiently. Strikingly, the improvement in time does not imply that the paths found are also shorter. In Fig. 1c, the stretch of human solutions is shown compared to the shortest paths. The stretch of a path *P* is computed as the ratio of the length of *P* to the length of the shortest path between identical starting and destination words. In the example of Fig. 1a, the stretch of the human path (green) is $\frac{5}{4}=1.25$ compared to the shortest possible path (red). Figure 1c shows that although human subjects improve in terms of the time needed to solve a task, the stretch of the paths they find stabilizes slightly below 1.2. Thus, the length of the human paths seems not to converge to the length of the shortest path (i.e., to stretch 1), and they always include some detours. A plausible explanation for this is that human subjects develop some kind of sub-optimal strategy through the course of the game and use this strategy to solve upcoming tasks. The improvement in time only means that the application of the same strategy becomes increasingly more effective. Nevertheless, how can we characterize the strategy in use?

**Figure 2:**
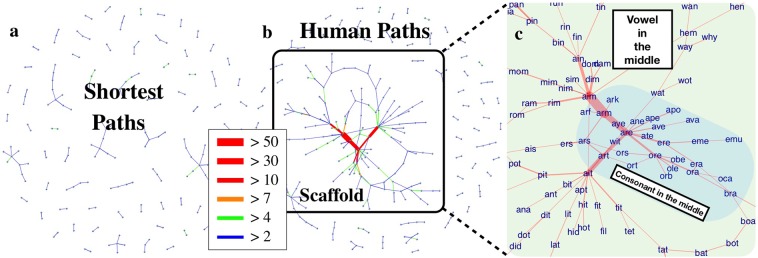
Structures behind human paths and shortest paths. Panel (**a**) shows how many times an edge is crossed after solving 1000 random tasks by using the shortest path between the source and target word. The almost homogeneous distribution of edge crossings suggests that the entity using these paths does not have any form of understanding or interpretation of the word-morph network; conversely, it mechanically picks paths. Human paths are quite the contrary. Panel (**b**) shows the edge crossings of a single human subject when solving the same 1000 random tasks. The human solution appears to be highly structured, suggesting that humans possess a characteristic concept of the word-morph network. The structure is very close to a pure hierarchy. There is a clear scaffold that guides navigation, consisting of red, orange and green edges with a high number of crossings. This scaffold shows that the human subject tends to simplify the problem and form a simpler and systematic, although not necessarily optimal, strategy. From the sides of the network, where a navigation task starts, the human subject tends towards the scaffold where a switch is performed to other sides of the network. How this particular scaffold is built up is quite specific. Panel (**c**) shows the words in the middle of the scaffold. “Aim”, “art”, “arm” and “are” depict words where consonants and vowels can be changed very effectively. In this case, the scaffold is used to switch between regimes of the network based on the location of vowels and consonants.

**Figure 3:**
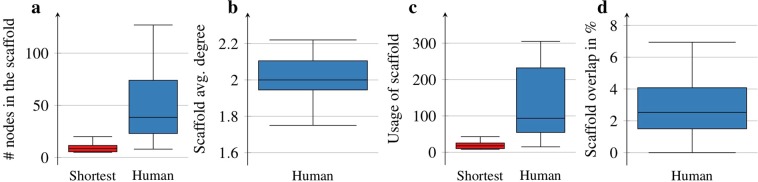
Properties of individual human scaffolds. Panel (**a**) shows the size of the human scaffolds compared to the shortest path case. The human subjects’ behaviour clearly deviates from the shortest path algorithm, as they form sizeable navigational scaffolds compared to shortest paths. The average degree of the scaffolds is close to approximately 2, as shown in panel (**b**); thus, the structure is very close to trees. Panel (**c**) confirms that the scaffold is heavily used by human subjects when completing the navigation tasks. We define usage simply as the sum of intersections between the subject’s paths and the scaffold. If we denote the solutions of the subject as *P*_1_, *P*_2_…*P*_*K*_, where *K* is the number of puzzles solved by the subject, then the usage of the scaffold *S* is computed as ${\sum }_{i=1}^{K}E({P}_{i})\cap E(S)$, where *E*(*P*_*i*_) denotes the set of edges contained in *P*_*i*_, while *E*(*S*) is the set of edges in the scaffold. Panel (**d**) shows that the individual human scaffolds are indeed “individual” as the observed overlaps between the subjects’ scaffolds is only 2.6% on average.

Panels **a** and **b** in Fig. 2 illustrate how differently an algorithm implementing shortest paths and a single human subject use the word-morph network to solve the navigational tasks. The plots show only edges traversed more than two times in the course of solving 1000 tasks. In the case of the shortest path algorithm, the usage of edges is homogeneous. The algorithm has no clear concept or deeper interpretation of the word-morph network and thus picks the paths mechanically without any sign of favouring specific regions of the network. The selected human subject behaves quite differently. The subject seems to have a clear concept of the network. The subject structures the network in a subjective manner by identifying various regions and places a larger emphasis on nodes and edges connecting these regions. A clear sign of this structuring is that from the human solution, a hierarchical scaffold structure is formed (see Fig. 2b for an example). To capture this behaviour, we focused on subjects highly engaged with the game, thus producing enough data to deeply examine the navigation strategy they use. We investigated subjects having more than 200 completed navigation tasks (9 subjects qualified for this). For these subjects, we processed all the solutions of the navigational tasks and assigned weights to the edges of the word-morph network reflecting how many times they were used in the solutions. We dropped the rarely used edges, for which the usage could be the result of randomly choosing the source and destination words. From the remaining graph, we took the largest component as the scaffold. In 90% of the cases, the scaffolds of the human subjects were at least two times larger in size compared to the random case, but in the majority of the cases, the human scaffolds were found to be an order of magnitude larger (see Panel **a** in Fig. 3).

Panel **b** of Fig. 3 shows that the average degree of the scaffolds is approximately 2 in the case of all subjects. This means that the scaffolds are tree-like connected sub-networks of the original word-morph network. This result is fully in line with the assumptions of existing hierarchical human navigational models^6,17,18,20^. Compared to shortest paths, the edges of the scaffolds are heavily used by the subjects (see Fig. 3c) with a very specific usage pattern. The scaffold has a definite core of a few nodes, between which the usage of the edges can exceed 50 in the particular example of Fig. 2b. This core behaves as a switching device among different parts of the network and abstracts the individual’s concept of the structure of the whole network. The scaffold is built up in a hierarchical, tree-like fashion, as edge utilization clearly drops when receding from the core. In the course of navigating between words, subjects use the scaffold as a guiding framework. Figure 2c shows the words residing in the scaffold. In this example, the network is clearly divided into regions based on the position of consonants and vowels in the words, and the core words are picked by the human subject in order to switch effectively among these regions. Our results show that although these individual scaffolds may have some similarities, every subject used a fairly unique set of nodes and edges forming their own hierarchical scaffolds (see Supplementary Fig. 1 for additional examples of personal scaffolds). This finding is readily supported by Fig. 3d, which shows the percentage of overlap between all possible pairs of scaffolds. The overlap for scaffolds *i* and *j* is computed according to the Jaccard index over the sets of edges: $\frac{E({S}_{i})\cap E({S}_{j})}{E({S}_{i})\cup E({S}_{j})}$, i.e., the ratio of edges present in both scaffolds (*E*(*S*_*i*_) denotes the set of edges contained in scaffold *i*) to the edges in the union of the scaffolds. Thus a network’s overlap with itself is practically 100%. One can see that in the case of the scaffolds of the subjects, the average of the overlap is very small, approximately 2.6%, and the maximum overlap is only 7%.

To quantify the statistical significance of the results regarding the scaffolds, we tested the null hypothesis that human paths can be explained by the shortest path algorithm. To test this hypothesis, we generated 500 solutions with the random shortest path algorithm over the same set of puzzles that the subjects solved. We found that the distribution of scaffold sizes and usage can be nicely estimated with a Weibull distribution (see Methods) in the case of all subjects. Table 1 shows the parameters of the Weibull distributions fitted to the scaffold sizes and usages plus the p-value indicating the tail probability that a scaffold of similar size and usage to the human solution could be derived from randomly chosen shortest paths. The p-values never exceed the alpha level of 0.05 and are extremely small in most of the cases, meaning that we have to reject the null hypothesis with high statistical significance. This substantiates the conclusion that the behaviour of the human subjects cannot be explained based on the shortest path algorithm.

**Table 1:** Statistical analysis of scaffold size and usage.

Parameters of fitting and p-values for scaffold sizes				Parameters of fitting and p-values for scaffold usages			
#	Wei. shape	Wei. scale	p-value	#	Wei. shape	Wei. scale	p-value
1	3.06	25.22	4.57E − 62	1	2.92	87.47	9.58E − 202
2	3.09	12.83	0.00E + 00	2	2.86	25.26	0.00E + 00
3	3.83	18.97	3.10E − 03	3	3.72	40.97	1.99E − 03
4	4.14	16.38	3.56E − 04	4	4.19	38.74	4.78E − 05
5	3.81	14.50	6.98E − 149	5	3.51	28.97	0.00E + 00
6	4.07	5.16	2.61E − 03	6	3.18	8.60	2.84E − 03
7	3.96	9.37	1.70E − 304	7	3.50	18.60	4.31E − 298
8	3.26	9.99	3.05E − 04	8	2.89	19.50	1.05E − 04
9	4.25	7.36	0.00E + 00	9	3.93	14.91	0.00E + 00

The null hypothesis is that the solutions of human subjects are random shortest paths. To test this hypothesis, we generated 500 solutions with the random shortest path algorithm over the same set of puzzles that the subjects solved. Parameters of the Weibull distributions fitted to the scaffold sizes (left panel) and usages (right panel) and the p-value referring to the null hypothesis are given for all the subjects.

The identification of the individual scaffold hierarchies as core switching devices in the human interpretation of the word-morph network poses an intriguing question: Why do we use them even after mastering our ability in the navigation task? Why do we tolerate sub-optimal paths through these scaffold hierarchies and not strive for shorter paths? Recall that detours in the subjects’ paths persisted even after completing 100 navigation tasks. We argue that the reason behind this is related to our information encoding and processing capabilities. In short, we build scaffold hierarchies while being satisfied with sub-optimal paths because this way we do not have to process every bit of information about a large and complex system, and we can get away with an interpretation that is an order of magnitude simpler. To show this, we use the following minimalist information-theoretic model inspired by our results above. The word-morph network is represented by a graph *G*(*N,E*) defining its nodes *N* and edges *E*.

For modelling human behaviour, we use a simple tree hierarchy as a scaffold for navigation. The construction of the hierarchy proceeds by picking the node with the highest closeness centrality^27^ and building the breadth-first search (BFS) tree emanating from it. This BFS tree will be used as the scaffold. Inspired by the information exchange algorithm well-fitted for hierarchically structured organizations^17^, we define human navigation based on the scaffold hierarchy as follows: (*i*) if the destination node is below the current node or its neighbours in the hierarchy, then we step to its closest superior or the destination itself provided that the destination and the current nodes are connected; (*ii*) if the destination node is not below the current node in the hierarchy, then we step to the current node’s direct superior in the hierarchy. As an analogy, this simple navigation mechanism captures that if somebody is my subordinate in the hierarchy or the subordinate of someone that I know, then I know who is the closest to them among my acquaintances. If I know nothing about the target, then I turn to my direct superior. Note that this extremely simple process models only a possible way of using a very artificial scaffold, the BFS tree. Our goal with analysing this simplified navigation process is to enable the information-theoretic analysis of the paths formed by the usage of scaffolds. Paths emanating from this simple model will clearly not match the paths used by any of the subjects for multiple reasons. First, although scaffolds built by humans are very similar to trees, they are not trees in many of the cases (see Fig. 3b). Second, human scaffolds vary subject by subject and have only an extremely small overlap across subjects (see. Fig. 3d) and with the BFS hierarchy.

To characterize the complexity of implementing the paths provided by the shortest path algorithm and human navigation, we approximate the required minimum information in every node to decide which next step to take towards all destinations in the word-morph network. Let us assign positive integers, i.e., 1, 2, 3…, as IDs to the nodes of the network. At each node *x*, we can represent the amount of information needed to make the right choice by a node table *T*_*x*_. At node *x*, this node table has |*N*|−1 entries (where |*N*| is the number of nodes in the word-morph network) belonging to all the nodes other than *x*, and each entry contains the ID of a neighbour to take next towards a given destination. For example, a node table *T*_5_ = (1, 2, 1, 2) tells us that at node 5, if we want to go toward node 1, 2, 3, 4, we should take nodes 1, 2, 1, 2 as next steps, respectively. This node table implicitly tells us that node 5 is connected to nodes 1 and 2 and that, in this example, the network has five nodes. Supplementary Note 1 provides a more detailed example of how to compute these node tables for a concrete network and set of paths. Now, tables ${T}_{x},\,\forall x\in N$ contain all the information required to implement the given paths between arbitrary pairs of nodes in the word-morph network. To approximate how many bits of information are needed to store these tables in memory, we compute their empirical Shannon entropy^28^, defined as ${H}_{0}({T}_{x})={\sum }_{c\in \Sigma }\frac{{n}_{c}}{n}\,\log \,\frac{n}{{n}_{c}}$ where Σ denotes the set of different numbers in *T*_*x*_, while *n* and *n*_*c*_ represent the length of *T*_*X*_ and the number of occurrences of each number $c\in \varSigma$ in *T*_*x*_, respectively. Then, $\frac{{\Sigma }_{x\in N}{H}_{0}({T}_{x})}{n}$ yields the global per node entropy to implement the paths. In Supplementary Note 2, we supply asymptotically optimal results for the empirical entropy of some well-known graph families.

**Figure 4:**
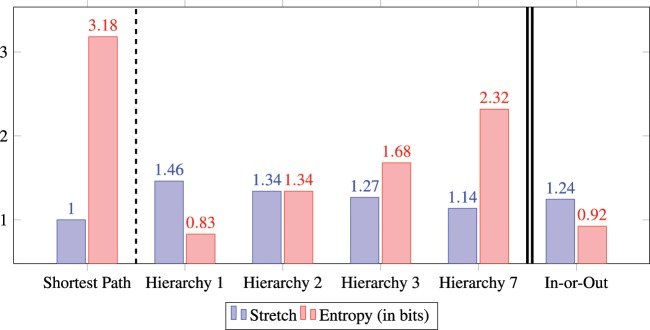
Comparison of stretch and entropy of various paths. Shortest paths clearly have a stretch of 1, but this optimality comes at a price of high entropy, i.e., a high memory requirement for storage. Hierarchies 1–7 show the very efficient stretch-entropy tradeoff if we memorize only 1–7 uplinks in the simplest BFS hierarchy. The decentralized In-and-Out hierarchy with one direct superior, based on the highest closeness centrality, is a sweet spot in this tradeoff space. This simulates the case when people know all subordinates in the network but remember only one superior closest to the centre of the network. It provides a realistic stretch, but the required entropy is an order of magnitude lower than that in the shortest path case.

In Fig. 4, the required information for implementing shortest paths and hierarchical paths in the word-morph network is shown. Shortest paths clearly have a stretch of one, but the price of this is a high entropy, as approximately 3.18 bits per node are required to store the shortest paths in the node tables (see the Shortest Path column on the left of Fig. 4). Navigation with the simple BFS scaffold has an order of magnitude less (approximately 0.83 bits per node) entropy (see the Hierarchy 1 column of Fig. 4), but hierarchically guided paths are much longer; they have a stretch of 1.46. Recall that our results with human subjects indicate a stretch slightly below 1.2. The hierarchy 2, 3 and 7 columns in Fig. 4 stand for a slightly modified version of the BFS hierarchy in which we do not have strictly 1 direct superior but can have links to at most 2, 3 and 7 superiors in the BFS tree, respectively. These hierarchies are no longer trees, but they are still as sparse as the human scaffolds. These modifications readily illustrate that there is a clear tradeoff between stretch and entropy. Having to remember more superiors reduces the stretch but surely increases the complexity. Nevertheless, with hierarchy 7, a stretch of 1.14 is achievable at the cost of only 2.32 bits of memory per node. These results readily illustrate that even the most rudimentary scaffold guiding navigation can achieve an effective stretch-entropy tradeoff. However, BFS scaffolds are constructed in a centralized fashion and rely upon global information about the network, which is not realistic. A more realistic decentralized scaffold with only one direct superior yields a sweet spot in this tradeoff space while being computable with local algorithms^29^. In this hierarchy, called In-or-Out, every node’s superior is the neighbour lying in the most central location in the network in terms of closeness centrality. This simple, local strategy can provide a very low stretch for an order of magnitude less entropy compared to shortest paths. This is because the In-or-Out hierarchy is aware of the neighbours’ centrality; thus, every node’s direct superior is a neighbour that is closest on average to any other node in the network. Interestingly, the In-or-Out hierarchy stretch is close to what we have observed with human subjects.

**Figure 5:**
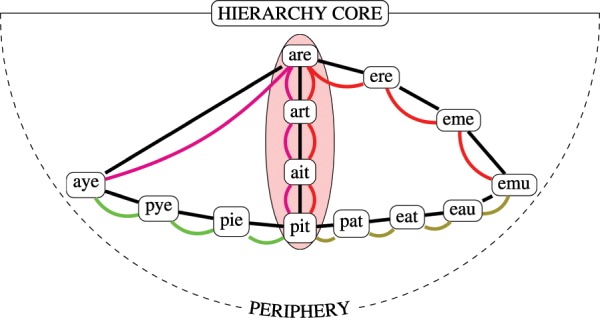
Shortest and hierarchically guided paths in the word network. Learning only the shortest paths between the words and and between and makes us conclude that the word *aye* is 7 nodes away from *emu*. However, with a hierarchical scaffold, a four-node path between *aye* and *emu* can be found even though both of the paths between *pit* and *aye* and between *pit* and *emu* are longer through the scaffold than through the shortest possible path.

**Figure 6:**
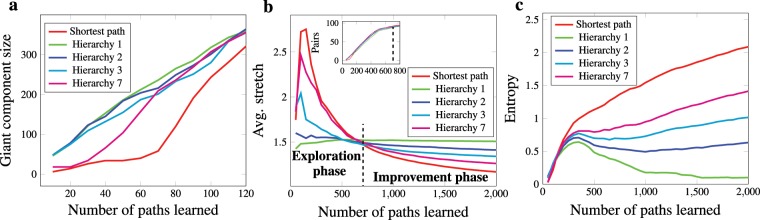
Learning curves in the word-morph network. Panel (**a**) shows the size of the giant component vs. the number of paths learned according to various learning scaffolds. Using the shortest paths as the scaffold yields sporadic knowledge about the network, especially in the initial steps of learning, since the size of the giant component is very low compared to the other scaffolds. The most integrated knowledge about the network is given by the simplest Hierarchy 1 in the initial steps of learning. The inset of panel (**b**) shows that after learning only approximately 700 paths, one can infer valid paths between 90% of all possible node pairs using either the shortest path or hierarchical scaffolds. In this exploration phase, learning based on shortest paths seems to be quite inefficient, as the stretch can even reach 3. In this phase, the simplest hierarchical scaffold yields the shortest established path on average. Only in the improvement phase, in which no significant new parts of the word-morph network are explored, is the relation is reversed. The entropy of the paths is shown in panel (**c**). The exploration phase shows no difference among the scaffolding schemes; however, in the improvement phase, the entropy of the hierarchical scaffolds is much lower compared to the shortest paths.

In addition to simplifying the process of navigation, scaffold hierarchies can boost learning the structure of a totally unknown network by observing its paths. To show this, we use a very simple incremental model where, in every step, we show a single path connecting randomly chosen nodes and compare the reconstructed network structure and the efficiency of navigation based solely on the given paths to the original network. Figure 5 illustrates the steps of this learning process for the cases in which we show paths according to shortest or hierarchical scaffolds from the word-morph network. In the first case, we show the shortest paths between the words “aye” and “pit” (green) and between “pit” and “emu” (olive), and based solely on this knowledge, one may implicitly deduce a path from “aye” to “emu” traversing 6 nodes. Alternatively, showing paths using a hierarchical scaffold yields somewhat longer paths (red). However, one can see that the newly gained path between “aye” and “emu” leads to a substantially shorter path requiring only 3 intermediate nodes. In Fig. 6, the integrity and the stretch and entropy footprint of the various learning scaffolds are shown when we continue simulating the learning process up to 2000 paths with a computer program (see Methods for details). In panel (**a**), the size of the giant component in the network reconstructed from the paths is shown as a function of learned paths. The shortest path scaffold provides only very sporadic knowledge about the network in the initial (0–120) learning steps, as the size of the giant component hardly grows with the number of learned paths. The most integrated knowledge is provided by the most simple scaffold of Hierarchy 1. In panel (**b**), we can clearly distinguish between two phases of the learning process. Until approximately 700 paths, rough exploration of the nodes and possible connections in the network occurs. According to the inset of the panel, by the end of this exploration phase, one can connect more than 90% of all possible node pairs in the case of all scaffolds. Using the shortest paths as learning scaffolds, we can find only very long paths in the exploration phase, as the average stretch can exceed even 3. Interestingly, if paths are picked according to a hierarchical scaffold, we can obtain paths with a lower stretch as the scaffold becomes increasingly simpler, i.e., the number of direct superiors decreases. In the case of the simplest one-superior case, the stretch is very stable at approximately 1.5. Therefore, in the exploration phase, one can learn reasonable paths much faster if paths are given according to a hierarchical scaffold. After the exploration phase, we do not explore new territories of the word-morph network; what we do is only improve our knowledge. In this improvement phase, the shortest path scaffold takes the lead over the hierarchical scaffolds, yielding the best stretch values. The price of being better in stretch is a higher entropy, as can be seen in panel (**c**): The entropy of the scaffolds is similar in the exploration phase; however, as the number of paths learned increases, the entropy of the simplest Hierarchy 1 scaffold starts to decrease substantially, while the shortest path one continues to increase almost linearly.

## Discussion

Although this study concentrates on a networked system, the underlying problem of human navigation in the word-morph network seems even more interesting in light of the fact that the current explanations of physical navigation tend to apply models considering the graph-like abstraction of the surrounding physical environment. In fact, there is an ongoing debate about whether we build a detailed cognitive map or a much simpler cognitive graph of the possible physical choice points^30,31^ inside our head. Furthermore, recent studies reported major correlations between the navigation and learning skills of humans^32,33^, while others went even further and investigated the possibility that navigation in cognitive spaces may lie at the core of any form of organized knowledge and thinking^34–36^. The word-morph network is a special mixed system over which navigation relies strongly on domain-general mechanisms since both spatial, manifested in the Hamming distance between words, and cognitive, i.e., the function and meaning of the words, dimensions contribute to the formation paths. Thus a promising speculation is that the identification of individual scaffolds guiding human navigation in the word-morph network may contribute to a better understanding of how humans structure, encode and navigate through cognitive spaces.

The empirical confirmation of individual scaffold hierarchies may also help resolve known anomalies in modelling human navigation behaviour in networks. Human paths over networks are reported to exhibit non-negligible memory^24,37,38^, which leads to problems when applying first-order Markov chains to approximate paths in spreading dynamics and community detection^24^. Individual scaffold hierarchies explain the source of these anomalies, as the next step of hierarchically guided paths clearly depends on nodes visited previously by the given individual. Building on the assumption of hierarchical scaffolds behind network paths, we may be able to refine higher-order Markov models, which may bring us closer to a better understanding of how real systems are organized and function.

## Methods

### Dataset

**Figure 7:**
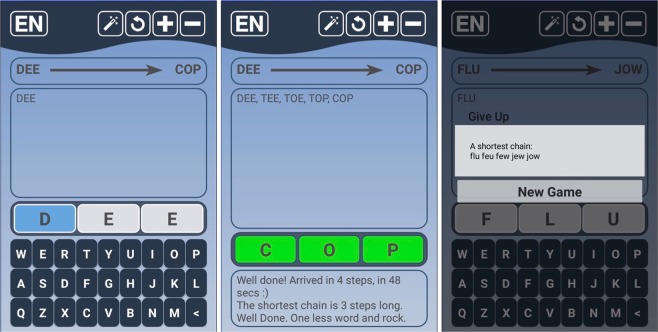
The main screen of the fit-fat-cat application.

For our study, we have used the dataset collected by a smartphone application called “fit-fat-cat” running on the Android platform. The dataset^11^ is published in Scientific Data, with the appropriate ethical consent. Here, we summarize the data collection process; for a detailed description of the experiment, consult^11^. The application is available from the Google Play store^26^. When a subject starts a navigational task, the source and destination words are generated randomly from the all possible three-letter English words. The source and destination words are displayed in a box (see Fig. 7). Below this box, the list of words that the subject visited so far in that particular task is shown. When starting a new task, the list contains only the source word. The subject can enter the consecutive words in a user-friendly manner by using a virtual keyboard of the phone. First, the subject selects the letter to change, then chooses the new letter with the keyboard. After changing a letter, the app automatically adds the new word to the list. In this way, the subjects can see which words they have already tackled when solving a particular navigation task. A task may end in three ways. If the subject reached the target word through such one-letter transformations, then the task is solved. In this case, the word becomes green-coloured to show the end of the task. Second, the subject can give up the task by pressing the “new game” button. In this case, the subject acquires the next task automatically. Finally, the subject can press the “magic wand” button. In this case, a possible (shortest path) solution of the task is shown before starting a new task. No matter how the task is ended, the list of words is anonymously submitted to our database stored in the cloud. Due to the scale of the experiment, we couldn’t control the external conditions under which the subjects carried our the solutions, apart from standard software checking of the validity of the subjects’ inputs. For more details, see^11^.

Detecting an individual scaffold requires a relatively high number of completed navigation tasks. Completing many puzzles can be a very tedious and repetitive task. Doing this in a single row (e.g. in a paid, controlled experiment during which the subject can concentrate from the beginning to the end) is arguably unfeasible. Luckily, 9 of the subjects found the game interesting enough to solve more than 200 puzzles. Thus it is not the number of subjects that are uniquely large in the dataset, but the number of paths collected from a single subject.

### Path filtering

Instead of focusing on the dynamic process of how we learn to navigate, i.e., how we learn an approximate picture of the network by exploration, we concentrate on the way people routinely choose paths in a network *after* they have developed their individual path selection strategy. In this steady state, subjects do not explore the network or wander around; they simply solve the puzzle by routine. To analyse this steady-state behaviour, we have to drop all unfinished paths, paths taking too much time to complete and loops from the dataset. Of the recorded 19828 paths, we dropped 8177 because they did not reach the target for some reason, 712 paths because the time to solve the puzzle was unusually large (>300 seconds), which raises the question of if the subjects concentrated on the puzzle, and only 352 paths (1.7% of the total paths) because they contained loops.

### Weibull fitting to the random shortest path algorithm

**Figure 8:**
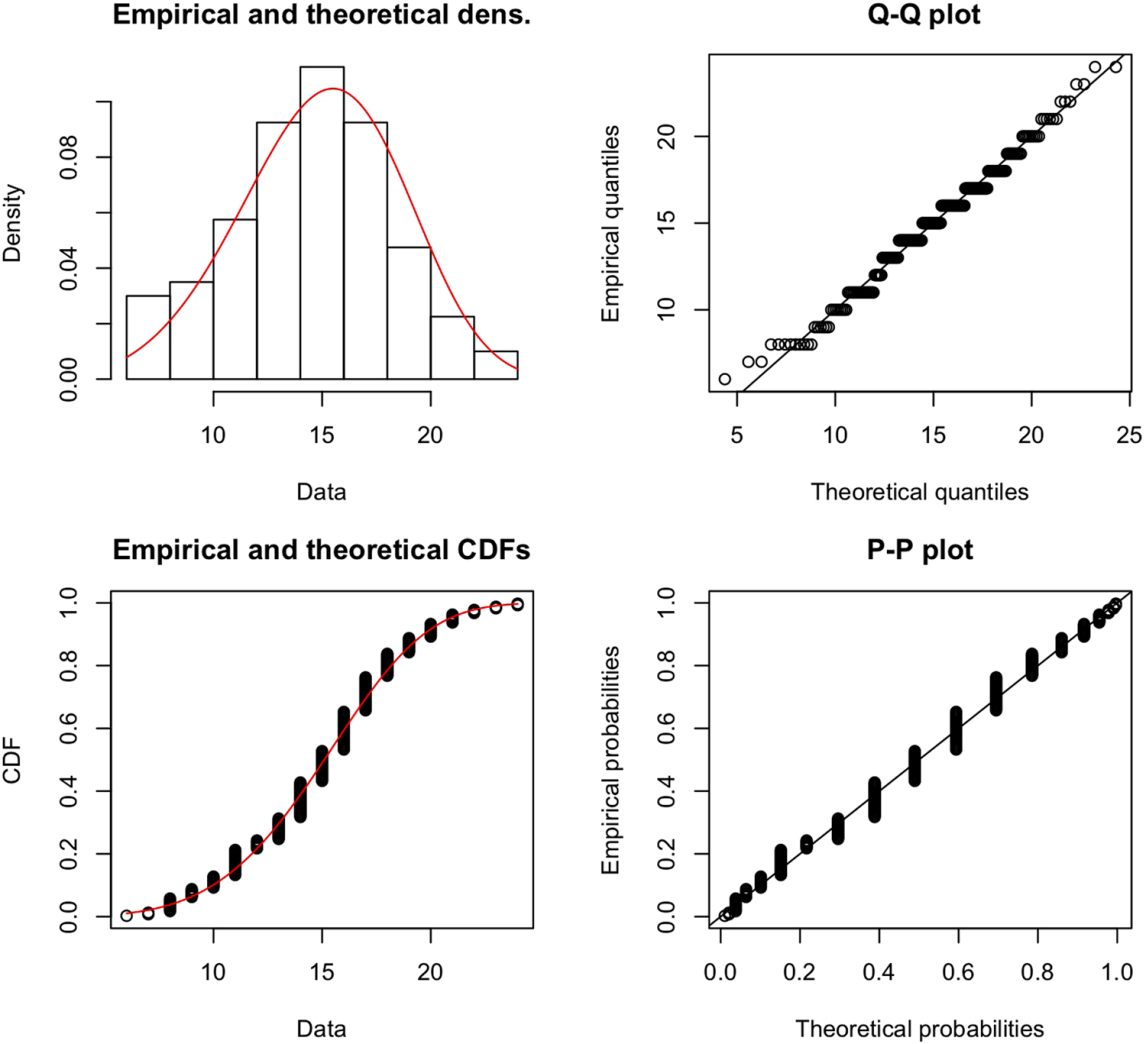
Goodness of fit of the Weibull distribution to the scaffold sizes given by the random shortest path algorithm over the puzzles of subject 4.

The scaffold sizes and usages of the random shortest path algorithm can be well-estimated with a two-parameter Weibull distribution. As an illustration, we verify the goodness of the fit for the puzzle set of subject 4 in Fig. 8. The results for the other subjects are highly similar.

### Computer simulations

For investigation of the incremental learning of a network via its paths, we have written a simulator in the Python programming language. In the beginning, the simulator reads the network *N*. After that, it iteratively picks random pairs from the network and computes the shortest and hierarchical paths between them according to the given BFS hierarchy. At each iterative step, the current knowledge about the network is the union of nodes and edges contained in the previous iterations. Therefore, at step *t*, the knowledge about the network is a graph *G*_*t*_(*V*, *E*); then, after adding a path *P*_*t*_, it is extended to ${G}_{t+1}={G}_{t}\cup {P}_{t}$ The simulator computes the required entropy and stretch of the paths in *G*_*t*_ compared to the shortest paths in *N* every 50 steps. We note, that we have run the simulations beyond 2000 paths but the relative positions of the stretch and entropy plots of the algorithms remains the same in that regime.

## Supplementary information

Supplementary information.

## Data Availability

The data supporting the findings of this study are available from the “fit-fat-cat” public Open Science Framework data repository^39^ and described in detail in^11^.
