# Supplementary material for: The role of detours in individual human navigation patterns of complex networks
Source: Sci Rep. 2020 Jan 24;10:1098. doi: 10.1038/s41598-020-57856-4 (PMC6981150; doi:10.1038/s41598-020-57856-4)
Supplement: Supplementary file 1 — Supplementary information. [file 41598_2020_57856_MOESM1_ESM.pdf]

Supplementary information for: The role of detours in individual human navigation patterns of complex networks

András Gulyás, József Bíró, Gábor Rétvári,
Márton Novák, Attila Kőrösi, Mariann Slíz and Zalán Heszberger
Corresponding author: András Gulyás (gulyas@tmit.bme.hu)

Supplementary Figures

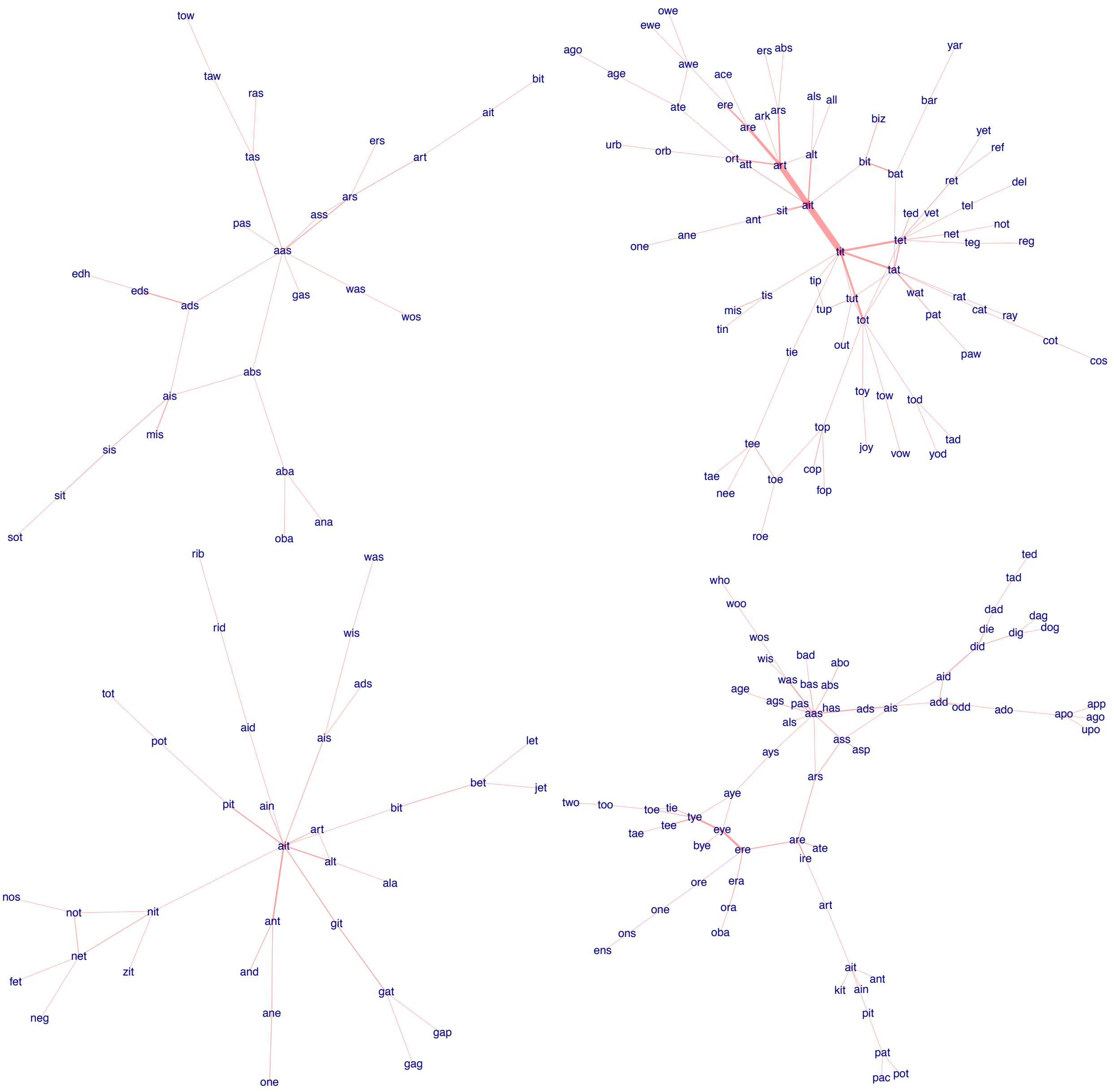

Supplementary Figure 1: Various human scaffolds.

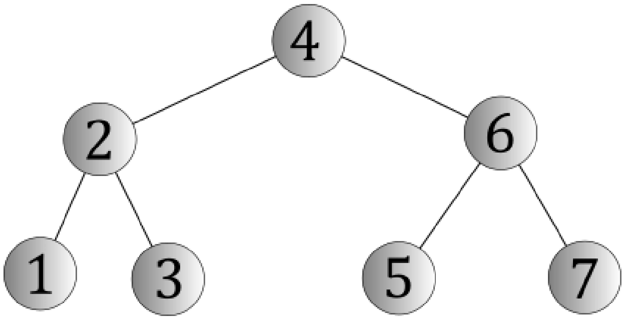

Supplementary Figure 2: Binary tree.

Supplementary Tables

Supplementary Table 1: Empirical entropy ($\bar{H}_0$) and compression ratio (η_0) of shortest path based node tables T_k necessary for navigation under the name independent model for various well-known families of graphs. In graphs where multiple shortest paths exist between many node pairs, for $\bar{H}_0$ we specify the lower and upper bound over an optimistic and a pessimistic choice for the next-hops.

Graph class	Empirical Entropy	η_0
complete graph	$\log n$	1
star	$\frac{\log n}{n}$	1
d-grid	$\frac{\log e}{2} \longleftrightarrow \log(2d)$	$\frac{\log e}{2\log(2d)} \longleftrightarrow 1$
path graph	$\frac{\log e}{2} \sim 0.72$	~ 0.72
d-torus	$1 \longleftrightarrow \log(2d)$	$\frac{1}{\log(2d)} \longleftrightarrow 1$
ring	1	1
d-hypercube	$2 \longleftrightarrow \log d$	$\frac{2}{\log d} \longleftrightarrow 1$
n-tree	$\frac{\log n}{n} \longleftrightarrow \frac{\log e}{2}$	NA

Supplementary Note 1 - Example computation of required memory

As an example, we show how to compute the memory requirement of a path selection algorithm in a simple binary tree shown in Supplementary Figure 2. When navigating in the tree, we always need to know in a given node, where to turn next to reach the destination. For example, if we want to go from 4 to 7, we must know in 4 whether to turn to 2 or 6. Then if we turn to node 6, then at node 6 we again need to know if we should go to 5 or 7 towards 7 (for the shortest path it is obviously 7).

So in each node we will have a table, showing where to go next towards all the destinations. For example the table of node 1 is extremely simple, because from node 1 we should go to node 2 no matter where we are heading to. So node 1's table is $(2, 2, 2, 2, 2, 2)$, because there are six possible destinations from node 1 and all are reached through node 2. Now checking the table of 4, to go to 1,2 and 3 we should turn to 2, and to the rest of the nodes (5,6,and 7) we should choose node 6. Along the same thoughts node 4's table is $(2, 2, 2, 6, 6, 6)$, while for and node 2 the table is $(1, 3, 4, 4, 4, 4)$.

The question is as to how much information we need to navigate between arbitrary points in the graph. Well, we should sum up all the information contained in the tables. This is where entropy comes into the picture. We can capture the amount of information if we compute their empirical entropy. The summation of the entropies gives the amount of memory (in bits) we need to be able to implement the given policy over the graph.

Supplementary Note 2 - Asymptotically Optimal Results for the Empirical Entropy of Some Well-known Graph Families

To compute the empirical entropy of the data structure necessary to navigate in special graph families we need to involve a few extra notations and modeling details. The choice network is defined by the graph $G(N, E)$. Let n denote the number of elements in N and let us introduce the routing function alphabet $\Sigma_v = [1, \delta_v]$ for each $v \in N$ summarizing the possible next hops in a node towards other nodes in the network. The next hops are collected into node tables $T_v, \forall v \in N$, listing them, as mentioned in Supplementary Note 1, in a fixed order. Alternatively Σ_v may contain the id's of *ports* (the closer end of edges) that connect node v to neighbors, the numbering of which being independent from the id's of neighboring nodes. Note that in this case the numbering of ports can be subjective, that is the id's of two endpoints of the same edge may differ in neighboring nodes. In the latter case, a better terminology for T_v is the *routing table*. For our current discussion both approach may suffice. To measure the information content of the node tables (that is to compute the empirical entropy of T_v) it is important to assume that the node id's or port numbers do not carry any more information apart from uniquely identifying the nodes or ports themselves. We could as well view this as if the ids were chosen by an adversary and so the memory at each node should be large enough to store any possible data to navigate around G arising over any permutation of ids. The above (so called name independent) setting maps naturally to the most simple empirical entropy model of information-theory: we can compute the node distribution in T_v as $n_i/n : i \in [1, \delta_v]$ for each port i of each $v \in N$, but no higher-order statistics, as those depend on the assignment of node ids that is beyond our control.

The following theorem gives a tight bound on the attainable compression (information content) of T_v in the name independent model.

Theorem 1. *Given a graph G on n nodes with each node assigned a unique id in $[1, n]$ under the* name independent model*, encoding the node tables $T_v : v \in N$ needs $nH_0(v) + o(n)$ bits memory, where*

$$H_0(v) = H_0(T_v) = \sum_{i \in [1,\delta_v]} \frac{n_i}{n} \log \frac{n}{n_i} \qquad (1)$$

and n_i denotes the number of times output port id $i \in [1, \delta_v]$ appears as a next-hop node/port in T_v.

Proof. Easily, $nH_0(v) + o(n)$ bits is enough to store T_v, so we only need to show that this is a lower bound as well. We observe that, depending on how the adversary assigns node ids, the next-hop node id 1 can appear at exactly $\binom{n}{n_1}$ positions in T_v, node id 2 can appear at $\binom{n-n_1}{n_2}$ positions, etc., putting the number of distinct routing functions we need to identify to

$$\binom{n}{n_1}\binom{n-n_1}{n_2}\cdots\binom{n-\ldots-n_{\delta_v-1}}{n_\delta} = \frac{n!}{\prod_{i=1}^{\delta_v} n_i!} \quad .$$

Using the Stirling formula $\ln n! \sim n \ln n - n + O(\ln n)$, we get that we need $\log \frac{n!}{\prod_i n_i!} = nH_0(v) + o(n)$ bits to identify each possible routing function. □

After deciding on the appropriate way of measuring the information content of any node table, our next step is to assemble $T_v, \forall v \in N$ themselves. For that purpose we have to lay down the expected properties of

the paths between nodes. In the following, we assume the node tables establishes shortest paths between any node pairs of a given graph G. Computing the empirical entropy of node tables for shortest paths in even simpler graphs is a difficult task, below we concentrate on some instances for which the problem *can* be solved: highly symmetric graphs from well-known graph families. Namely, we stick to the simplest possible case of shortest-path routing over unit cost graphs. The analysis will be for the average case: for each graph class we specify the average (over all nodes) empirical entropy $\bar{H}_0 = {}^1/_n \sum_v H_0(v)$ and the compression ratio $\eta_0 = {}^{n\bar{H}_0}/_{\bar{I}}$ where $\bar{I}$ denotes the average information-theoretic lower bound and $n\bar{H}_0$ is the mean routing table size in bits. The results are in Supplementary Table 1.

Full-meshes (complete graphs) and hup-and-spoke networks (stars) are amongst the most renowned graph topologies. In the name independent case, obviously, we get no compression at all for these topologies.

A d-dimensional grid (or mesh) is a graph whose natural embedding into the Euclidean space $\mathbb{R}^d$ forms a d-dimensional tiling and a path-graph is a one-dimensional grid; torii are "wrap-around" grids and a ring is a one-dimensional torus; and the nodes of a hypercube in an Euclidean-embedding form the corners of a d-cube. These graphs offer multiple shortest paths between most node pairs and, as it turns out, the way we select from these greatly affects the entropy bounds. Consider, for instance, hypercubes and suppose first that we control next-hop selection. Then, any node can reach half of the nodes on some shortest path via one of its edges, and half of the remaining nodes via another one, etc., and choosing those shortest paths $H_0 \sim 2$ for the node tables. If, however, next-hop selection is random, then starting from any edge "covers" roughly the same number of nodes so $H_0 = I = \log d$. Accordingly, under the name independent model an optimistic next-hop selection results very small constant empirical entropy and significant compression, while the pessimistic choice yields no compression at all for those graphs. Observe that both strategies tend to keep topologically close nodes close to each other in the address space.

Our last graph family, the trees, are cycle-free graphs. The respective space bounds depend on the actual tree topology, but the best bounds arise on stars and the worst cases are path-graphs. Still, every tree admits infinite compression over a post-order enumeration of the nodes.

Finally, we note that, contrary to what is available in the literature, the above characterizations are tight; the entropy bound is not just a limit on the compressibility of the node tables but it also betokens that *no routing scheme can navigate a graph in smaller space*, even if that scheme does not use node tables at all. This shows the power of an information-theoretic approach over a case-by-case analysis.
